# *Terminalia brownii* Fresen: Stem Bark Dichloromethane Extract Alleviates Pyrogallol-Induced Suppression of Innate Immune Responses in Swiss Albino Mice

**DOI:** 10.1155/2023/9293335

**Published:** 2023-02-21

**Authors:** Jane Wanja Mbiri, Kenneth Ogila, Patrick Kisangau, Michael Gicheru

**Affiliations:** ^1^Department of Life Sciences, South Eastern Kenya University, Kitui, Kenya; ^2^Department of Zoology, Jomo Kenyatta University of Science and Technology, Juja, Kenya; ^3^Department of Zoology, Kenyatta University, Nairobi, Kenya

## Abstract

*Terminalia brownii* is widely used in folklore medicine and has diverse biological activities. However, its effect on the immune system is yet to be studied. Therefore, our study evaluated the immunomodulatory effect of *T. brownii* on nonspecific immunity. Innate immunity is the initial defence phase against pathogens or injuries. Dichloromethane plant extracts were tested on female Swiss albino mice and Wister rats. The effect of the extract on innate immunity was assessed via total and differential leukocyte counts, tumor necrosis factor-alpha, and nitric oxide production by mouse macrophages. The 3-(4, 5-dimethyl thiazolyl-2)-2, 5-diphenyltetrazolium bromide assay was employed for viability testing. Phytochemical profiling was carried out using gas chromatography-mass spectrometry, while toxicity studies were carried out following the Organization for Economic Cooperation and Development guidelines. Our results demonstrated that administration of *T. brownii* stem bark dichloromethane extract to pyrogallol-immuno compromised mice significantly (*p* < 0.05) increased total and differential leukocyte counts compared with the control. The extract showed no adverse effect on the viability of Vero cells and macrophages and significantly (*p* < 0.05) augmented tumor necrosis factor-alpha and nitric oxide production. Hexadecanoic acid, linoleic acid, octadecanoic acid, squalene, campesterol, stigmasterol, and *β*-sitosterol, all of which stimulate, were identified in the extract. The extract did not cause any death or toxic signs in rats. In conclusion, *T. brownii* dichloromethane extract has an immunoenhancing effect on innate immune responses and is not toxic. The observed immunoenhancing impact of the extract was attributed to the presence of the identified compounds. The results of this study provide crucial ethnopharmacological leads towards the development of novel immunomodulators for managing immune-related disorders.

## 1. Introduction

Innate immunity is the initial phase of defence against viral and bacterial infections and sterile inflammation [1]. It is a nonspecific defence mechanism that the host uses after an antigen encounter, instantly or within a few hours. However, innate immunity does not have immunologic memory. Thus, it cannot recognise the same pathogen if the body encounters it in the future [2]. Innate immunity has different types of protective barriers, including anatomic (mucous membrane and skin), physiologic (chemical mediators, low pH, and temperature), phagocytic and endocytic, and inflammatory barriers [2]. Nonspecific immunity comprises cellular and humoral components that identify, inactivate, and kill invading pathogens [3]. The cellular part includes phagocytes (neutrophils and macrophages), eosinophils, basophils, monocytes, mast cells, dendritic cells, innate lymphoid cells, and natural killer cells [2, 4]. However, the humoral component includes complement proteins, collectins, and antimicrobial peptides, among other elements [5]. Primary innate immune responses involve the recruitment of effector cells to the site of injury or pathogen invasion, and inflammation, through the production of chemokines and cytokines. The produced cytokines, tumor necrosis factor (TNF), interleukin 6 (IL-6), and interleukin 1 (IL-1), also trigger local cellular responses to injury or infection and fever development [2].

Recent research has demonstrated plants as promising sources of immunomodulators [6–8]. This is due to their diverse biological activities, fewer side effects, easy availability, and affordability [9–15]. Numerous phytochemicals have been approved for clinical trials, and others, including quercetin and resveratrol, among others, have undergone rigorous clinical trials [16]. The most recent achievement in this field is the approval of an immunomodulator, colchicine, an alkaloid isolated from *Colchicum autumnale*, by the Federal Drug Administration in 2009 for treating acute gout flares and Familial Mediterranean fever [16]. Several Terminalia species, including *T. arjuna, T. bellirica, T. catappa,* and *T. chebula*, modulate immune responses [17–27]. The modulation of the immune system encompasses any adjustment of immune responses, including inhibition or stimulation of its function [28]. Stimulating immune responses is desirable for patients with a compromised immune system, including patients with HIV and AIDs. Alternatively, immunosuppression alleviates aggressive pathological immune responses, including the rejection of transplanted organs, inflammatory diseases, and autoimmune disorders, among others [19]. Pyrogallol is a commercially available polyphenol [29]. It suppresses immune responses through the elicitation of oxidative stress [30].

*Terminalia brownii* is a crucial medicinal plant belonging to the family Combretaceae. It is native to Uganda, Tanzania, Sudan, the Democratic Republic of Congo, Kenya, Somalia, Ethiopia, and Eritrea [31]. The plant is used in traditional medicine for managing various ailments, including yellow fever, diabetes, kidney disorders, diarrhea, rheumatism, gastric ulcers, allergy, wounds, back pain, stomachache, fever, malaria, and cough bronchitis, among others [32]. *Terminalia brownii* has a wide array of biological activities, including anticancer, antimalarial, antipyretic, antinociceptive, preneoplastic lesion prevention, antidiabetic, and antimycobacterial, among others [33–39]. Despite *T. brownii* being widely used in folklore medicine and its immense biological activities, its effect on innate immunity remains unclear. Therefore, this study evaluated the effect of *T. brownii* stem bark dichloromethane (DCM) extract on innate immunity.

## 2. Materials and Methods

### 2.1. Plant Materials

**Figure 1:**
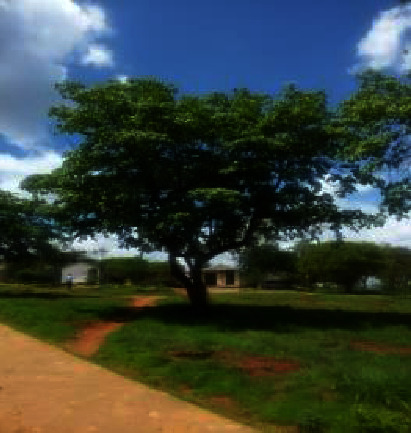
A mature *T. brownii * tree.

A stem bark sample of *T. brownii* tree (Figure 1) was acquired from Kitui, Kenya (1.3099°S 37.7558°E; about 152 km from Nairobi) in May 2021, aided by a native herbalist. Botanical verification was conducted at the East African Herbarium situated at the National Museums of Kenya, and a voucher specimen was deposited there (accession number: JWM001). The plant sample was taken to the animal breeding and experimentation facility at Kenyatta University for preparation and bioassays. The sample was rinsed using running tap water, reduced into tiny shreds, and shade-dried till thoroughly dried and powdered [40]. 

### 2.2. Extraction of Plant Materials

Extraction was carried out using dichloromethane (DCM) (Shandong Arctic Chemical Co. Ltd, Shandong, China). Three litres of DCM were added to 1500 g of the samples' powder. An aspirator pump, VE-11; Pfeiffer Vacuum, Inc., Nashua, NH, was employed to filter the extracts. The extracts were concentrated using a RE-501 rotary evaporator (Zhengzhou Keda Machinery and Instrument Equipment Co., Ltd, China) in vacuo. The extracts were stored at 4°C [41].

### 2.3. Experimental Animals

The Kenyatta University Animal Care and Use Committee (PKUA/005/005) authorized this study. Female Swiss albino mice (7–9 weeks old; 25–30 g) were used for immune response studies. According to [42], mice have played a significant role in many vital advances in immunology; hence, they are used in this study. Female mice were chosen for this study because they mount more robust immunological responses than males [43, 44]. On the other hand, female Wistar rats (2-3 months old; 140–180 g) were used for toxicity studies as guided by the OECD guidelines [45, 46]. The experimental animals were purchased from the animal breeding and experimentation facility at Kenyatta University. They were nourished with standard rodent pellets (Specialty feeds, Glen Forrest, West Australia) and water *ad libitum*. The animals were allowed to acclimate to laboratory conditions for a week before the bioassay tests. The composition of the standard rodent pellets is shown in Table 1 [47].

### 2.4. Evaluation of the Immunomodulatory Activity of *T. brownii* Stem Bark DCM Extract

The immunomodulatory activity of *T. brownii* DCM extract was assessed by evaluating total and differential leukocyte counts, nitric oxide (NO), and Tumor Necrosis Factor-alpha (TNF-*α*) production by murine macrophages.

#### 2.4.1. Effect of *T. brownii* Stem Bark DCM Extract on Total and Differential Leukocyte Counts

*(1) Experimental Design*. The animals were weighed and grouped into six groups, five animals in each. All treatments were administered orally, and the treatment period was two weeks. The first group received the vehicle, 2.5% dimethyl sulfoxide (DMSO) (Gaylord Chemical Company, LLC, Tuscaloosa, AL USA), in distilled water. The second group, negative control, was given 50 mg/kg BW pyrogallol (immunosuppressant) for the first seven days and then the vehicle until day 14. The third, fourth, fifth, and sixth groups received 50 mg/kg BW pyrogallol (Jiurui Biology Chemistry Limited, China) for seven days and 20 mg/kg BW levamisole (GNH, India), 50 mg/kg, 100 mg/kg, and 150 mg/kg extracts, respectively, from day 8 to day 14. On the 15^th^ day, blood was drawn from the tail vein to analyze total and differential leukocyte counts [48].

#### 2.4.2. Effect of *T. brownii* Stem Bark DCM Extract on NO Production by Murine Peritoneal Macrophages

*(1) Macrophage Isolation*. Newborn calf serum (NBCS), Life Technologies, US, was employed to trigger the release of peritoneal macrophages. Seventy-two hours afterward, peritoneal effusions were collected in Roswell Park Memorial Institute (RPMI) 1640 culture medium (Wuhan Servicebio Technology Limited, China). The outflows were centrifuged at 1000 rpm for 20 min, and RPMI 1640 culture medium was employed to rinse the cells twice. RPMI 1640 medium enhanced with L-glutamine (2 mM), gentamicin (0.04 mg/ml), 10% newborn calf serum, and penicillin 100 *μ*/ml were used to resuspend the cells [49]. Cell viability was assessed using methylene blue assay; viability was found to be above 95%.

*(2) Effect of T. brownii Stem Bark DCM Extract on Vero Cells and Macrophage Viability*. Viability tests were conducted via the MTT assay using 96 well culture plates [50]. Vero cells were procured from the American Type Culture Collection (ATCC), Manassas, Virginia. 100 Â*μ*L RPMI 1640 culture medium augmented with 10% fetal bovine serum was pipetted into all wells. A hundred microlitres of the extracts (3 mg/ml) were introduced into the culture plates' bottom row (row H) in triplicates separated by blanks. Levamisole (1 *μ*g/mL) was used as a positive control in assessing TNF-*α* and nitric oxide production by macrophages. Serial dilutions (two-fold) of the extracts were conducted up from row H to row C, and the excess (200 *μ*l) solution was disposed off. Rows B and A were left as the control wells.

The plates were incubated in a carbon dioxide (CO_2_) incubator for 24 h. After incubation, supernatants were drawn out to assess NO and TNF-*α* production by macrophages. Forty microlitres of MTT were added to all wells, and the plates were incubated for four hours. Culture media and MTT were pipetted out of the wells, DMSO (100 *μ*l) was pipetted into the wells, and plates were incubated further for 20 min. Enzyme-linked immunosorbent assay (ELISA) reader (Biobase Industry, China) was used to read the plates at 570 nm. The following formula was used to compute the percentage viability:

(1) $$\begin{array}{c} Cell\;viability=\left(\frac{Sample's\;or\;standard\;drug's\;absorbance}{Control's\;absorbance}\right)*100\text{.} \end{array}$$

*(3) Evaluation of NO Production*. The Griess reagent system was employed to estimate the levels of the NO produced by macrophages [51]. 50 *μ*l of the supernatants were admixed with 50 *μ*l Griess reagent in 96 well plates and incubated at room temperature for 10 min. An ELISA reader (Biobase Industry, China) was used to obtain absorbance values at 570 nm. A 0–100 *μ*M nitrite standard reference curve was generated and used to assess the concentration of nitrites.

#### 2.4.3. Effect of *T. brownii* Stem Bark DCM Extract on the Production of TNF-*α* by Mouse Macrophages

Mouse-specific Th1/Th2 cytokine Cytometric Bead Array (CBA) kit (BD Biosciences, US) was used to determine levels of TNF-*α* in macrophage culture supernatants as directed by the manufacturer. 50 Â*μ*L of bead populations with distinctive fluorescent extents, conjugated with analyte-specific antibodies, were added to 50 Â*μ*L of the samples and incubated for 90 min at room temperature. A standard curve was generated for the analyte. Samples and standards were rinsed to remove any detached components. Phycoerythrin-layered detection antibodies were added, incubated for an hour, and rinsed off. Fluorescence-activated single cell sorting (FACS)-Calibur flow cytometer (BD Biosciences, US) was used to quantitate the fluorescent signals generated. A Flow Cytometric Analysis Program (FCAP) software (BD Bioscience, US) was employed to establish the concentration of the analyte [52, 53].

### 2.5. Acute Toxicity Study

An acute toxicity study was conducted as outlined in the OECD guidelines for the acute oral toxicity up-and-down procedure [46]. A limit test was performed since aqueous and methanol extracts *of T. brownii* bark were previously reported to cause no death or toxicity at 2,000 mg/kg BW dosage in mice [39]. Female Wistar rats were divided into normal and test groups. A gavage needle was used to give the treatments as a single dose. Rats were fasted overnight, weighed, and treated. The animals further fasted for four hours. The control group received 2.5% DMSO in distilled water, while the test group received stem bark DCM extract of *T. brownii* at 2,000 mg/kg BW. Rats were observed for death and toxicity signs, including lethargy, coma, sleep, diarrhea, salivation, convulsions, tremors, and changes in the skin, fur, eyes, and mucous membrane. The body weights of the rats were retaken on days 7 and 14. After the study period, the animals were placed in a desiccator containing diethyl ether, and after they died, their carcases were incinerated.

### 2.6. Subacute Toxicity Study

This study followed the OECD guidelines for the repeated dose 28-day oral toxicity study in rodents [45]. Female Wistar rats were categorised into four groups. The first group was the control and received 2.5% DMSO in distilled water. The second, third, and fourth groups received *T. brownii* stem bark DCM extract at 300, 520, and 900 mg/kg BW dosages, respectively. A gavage needle was used to administer the treatments daily for 28 days. Rats were observed for death or toxicity signs daily; body weights were taken and recorded weekly. On day 29, the animals were weighed and sacrificed. Blood was drawn through cardiac puncture for haematology and biochemistry analysis. Body organs were also harvested, and their weights were taken.

#### 2.6.1. Analysis of Biochemical and Hematological Parameters

Blood samples for biochemistry analysis were put in Eppendorf tubes, while samples for haematology analysis were put in ethylenediaminetetraacetic acid (EDTA) tubes. The blood was centrifuged for 10 min at 3000 rotations per minute to acquire serum for biochemistry analysis [54]. Parameters assessed in both biochemistry and haematology are as listed by OECD guidelines [45].

### 2.7. Phytochemical Profiling of *T. brownii* Stem Bark DCM Extract

Gas chromatography-mass spectrometry (GC-MS) analysis of the plant extracts was carried out as described by [55] with a few modifications. A hundred milligrams of the sample were weighed, and 1 mL DCM was added. The mixture was vortexed for 10 s, sonicated for 10 min, and centrifuged for 5 min at 14,000 rpm. The supernatant was filtered and diluted to prepare 100 ng/*μ*L. Analysis was carried out on four replicates. The GC-MS instrumentation included a TRACE GC Ultra Gas Chromatograph conjoined with a Thermo mass spectrometer detector. The GC-MS system was fitted with a TR-5 MS column. Helium was used as the carrier gas and maintained at a flow rate of 1.0 ml/min and a 1 : 10 split ratio. Mass spectra were acquired via electron ionization (70 eV), using an m/z 40–450 spectral range. Quantification of the compounds was by the metabolites as distinguished by the mass spectrometer.

The detected compounds were identified using the AMDIS software by the retention times and mass spectrum analogous to standards (where available), and the National Institute of Standards and Technology (NSIT) database. Comparison with spectra of known compounds in the literature was also used to identify the detected compounds.

### 2.8. Data Analysis

GraphPad Prism 8 statistical software was used for data analysis. One-way analysis of variance (ANOVA) was carried out to assess the differences between groups. For mean values separation, Tukey's post hoc test was conducted. Values at *p* < 0.05 were considered statistically significant.

## 3. Results

### 3.1. Sample Powder and Plant Extract Yield

Four kilograms of fresh stem bark sample of *T. brownii* yielded 2.95 kgs of sample powder. Dichloromethane produced 28 g extract from 1500 g of *T. brownii* stem bark powder sample.

### 3.2. Effect of *T. brownii* Stem Bark DCM Extract on Total and Differential Leucocyte Counts

*Terminalia brownii* DCM extract enhanced the innate immunity of pyrogallol-immunosuppressed mice. The extract significantly increased total white blood cells (WBCs), neutrophils, lymphocytes, monocytes, eosinophils, and basophils count in the extract-treated mice compared with the negative control group (*p* < 0.05) (Table 2). The extract at 50, 100, and 150 mg/kg BW dosages produced a dose-dependent response in the increase of neutrophils. However, the effect of the extract at dosages 50 and 100 mg/kg BW on total WBCs, lymphocytes, eosinophils, and basophils was comparable (*p* > 0.05). The standard drug, levamisole, demonstrated a significantly (*p* < 0.05) higher activity in the augmentation of leukocyte counts compared to the extract at a dosage of 150 mg/kg BW (Table 2).

### 3.3. Effect of *T. brownii* Stem Bark DCM Extract on the Viability of Vero Cells and Macrophages

*Terminalia brownii* DCM extract showed no toxicity towards Vero cells; the extract at the highest dosage resulted in viability beyond 50% (Table 3). The extract produced a dose-dependent response at the tested concentrations (Table 3), and there was a significant (*p* < 0.05) difference between the activity of the extract at all concentrations and the control (Table 3).

The dichloromethane extract of *T. brownii* was not toxic to macrophages, as shown by the above 50% viability produced by its highest concentration (Table 3). The extract had a significant (*p* < 0.05) different effect on macrophage viability from dosage 187.5 *μ*g/ml to 3000 *μ*g/ml (Table 3). Furthermore, compared to the control, DCM extract at all tested doses had a significantly different effect on the viability of macrophages (*p* < 0.05) (Table 3).

### 3.4. Effect of *T. brownii* Stem Bark DCM Extract on NO Production by Mouse Macrophages

The dichloromethane extract of *T. brownii* augmented NO production by macrophages obtained from pyrogallol-immunosuppressed mice. The extract, from dosage 187.5 *μ*g/ml to 3000 *μ*g/ml, produced a significant (*p* < 0.05) difference in the production of NO compared to the untreated cells (Table 4). *Terminalia brownii* DCM extract at 375, 750, 1500, and 3000 *μ*g/ml dosages demonstrated a dose-dependent response. Furthermore, there was a significant (*p* < 0.05) difference in the activity of the extract at the highest dose, 3000 *μ*g/ml, and the standard drug (Table 4).

### 3.5. Effect of *T. brownii* Stem Bark DCM Extract on TNF-*α* Production by Mouse Macrophages

Dichloromethane extract of *T. brownii* increased the production of TNF-*α* by murine peritoneal macrophages acquired from pyrogallol-immunocompromised mice. The extract, at the tested concentrations, produced significantly (*p* < 0.05) elevated levels of TNF-*α* compared with the control (Table 5). Moreover, there was a significant (*p* < 0.05) difference between the activity of the extract at the highest dosage compared to the standard drug (Table 5). The extract at 187.5, 375, 750, 1500, and 3000 *μ*g/ml dosages produced a dose-dependent increment in TNF-*α* levels (Table 5).

### 3.6. Acute Toxicity

#### 3.6.1. Effect of *T. brownii* Stem Bark DCM Extract on the Body Weights of Female Wistar Rats

*Terminalia brownii* DCM extract did not affect the body weights of the experimental rats throughout the study period; extract-treated rats had comparable (*p* > 0.05) body weights with the control (Table 6).

#### 3.6.2. Effect of *T. brownii* Stem Bark DCM Extract on the Behaviour and Overall Appearance of Female Wistar Rats

*Terminalia brownii* DCM extract did not affect the behaviour and general appearance of the experimental animals (Table 7).

### 3.7. Subacute Toxicity

#### 3.7.1. Effect of Stem Bark DCM Extract of *T. brownii* on Female Wistar Rats' Body Weights

*Terminalia brownii* stem bark DCM extract, at all tested dosages, did not affect the body weights of the experimental animals; extract-treated rats had comparable (*p* > 0.05) body weights with the control from weeks one to four (Table 8).

#### 3.7.2. Effect of *T. brownii* Stem Bark DCM Extract on Female Wistar Rats' Organ Weights

Stem bark DCM extract of *T. brownii*, at all tested dosages, did not cause changes in the organ weights of the rats. Relative organ weights of extract-treated rats were comparable (*p* > 0.05) to those of the normal control group (Table 9).

#### 3.7.3. Effect of *T. brownii* Stem Bark DCM Extract on Female Wistar Rats' Hematological Parameters

Dichloromethane extract of *T. brownii* stem bark, at all tested dosages, did not alter levels of the assessed hematological parameters; levels of the parameters were similar (*p* > 0.05) between the extract-treated animals and the control (Table 10).

#### 3.7.4. Effect of T. brownii Stem Bark DCM Extract on Female Wistar Rats' Biochemical Parameters

The extract, at all tested dosages, did not affect levels of the analyzed biochemical parameters; levels of the parameters were similar (*p* > 0.05) between extract-treated rats and the control (Table 11).

### 3.8. Phytochemical Profile of *T. brownii* Stem Bark DCM Extract

**Figure 2:**
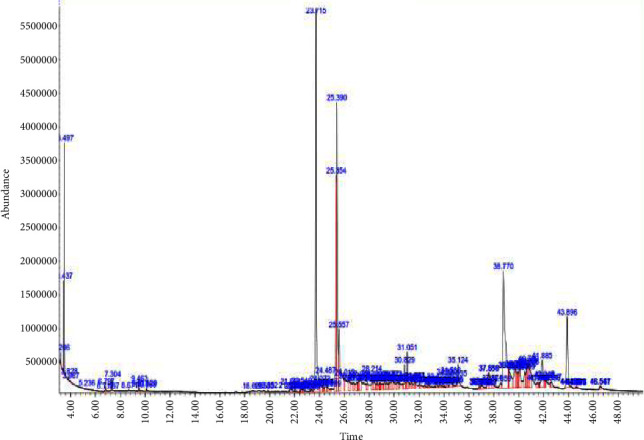
GC-MS chromatogram of *T. brownii* DCM stem bark extract.

Gas chromatography-mass spectrometry analysis of stem bark DCM extract of *T. brownii* showed fatty acids (hexadecanoic acid, linoleic acid, and octadecanoic acid), a triterpenoid (squalene), and steroids (campesterol, stigmasterol, and beta-sitosterol) (Table 12). Hexadecanoic acid was the most abundant, while stigmasterol was the least (Figure 2; Table 12).

## 4. Discussion

Our study evaluated the immunomodulatory effect of *T. brownii* stem bark DCM extract on innate immune responses by assessing leukocyte counts, TNF-*α*, and NO production by macrophages harvested from pyrogallol-immunocompromised mice. The findings of our study demonstrate the ability of stem bark DCM extract of *T. brownii* to revert immunosuppression of innate immune responses caused by pyrogallol. The extract augmented total and differential leukocyte counts, TNF-*α*, and NO production by mouse macrophages compared with the control. These results demonstrate the ability of *T. brownii* stem bark DCM extract to stimulate innate immunity, making *T. brownii* a potent candidate for developing new immunostimulators that can be used to manage immune-related conditions.

Treatment of mice with pyrogallol resulted in the production of significantly lower numbers of total and differential leukocytes compared with the control. However, stem bark DCM extract of *T. brownii* inversed this reduction and augmented total and differential leukocyte counts in the blood of extract-treated mice. Leukocytes are vital cells in innate immune responses. They protect the body through various mechanisms, including the release of cytokines, phagocytosis, and the presentation of antigens to T cells [56]. Therefore, reduced numbers of leukocytes in the body lead to opportunistic infections since the body is not well equipped to fight invading pathogens. Hence, it is critical to maintain leukocyte counts within normal ranges to counter the immunosuppressive status [57]. The increased leukocyte numbers in extract-treated mice could be attributed to the capacity of the extract to induce the production of WBCs in the bone marrow or augment the production of leukocytosis mediators [58].

The compounds identified in the stem bark DCM extract of *T. brownii* augment different elements of nonspecific immunity. Among the compounds identified in the extract, only squalene and *β*-sitosterol enhance leukocyte count in rodents. Squalene significantly increased the previously reduced leukocyte counts in 3‐methylcholanthrene-intoxicated rats [59]. *β*-sitosterol administered to mice significantly augmented lymphocyte counts compared to the control [60]. The ability of *T. brownii* stem bark DCM extract to increase leukocyte counts in immunocompromised mice can therefore be attributed to squalene and *β*-sitosterol.

The results of our study demonstrated that immunosuppression impaired the ability of mouse macrophages to produce a critical proinflammatory mediator, NO. However, stem bark DCM extract of *T. brownii* stimulated macrophages to produce higher NO levels. This result shows the ability of *T. brownii* DCM extract to elicit innate immune responses, especially in immunocompromised subjects, by classically activating macrophages [61]. Stimuli, including fungal and bacterial components, chemical mediators, cytokines, and bioactive plant compounds, can classically activate macrophages to make and release cytotoxic mediators, including NO and pro-inflammatory cytokines that help eliminate invading pathogens [51, 61].

We attributed the capacity of *T. brownii* DCM extract to augment the NO production by mouse macrophages to the presence of hexadecanoic acid, octadecanoic acid, linoleic acid, and stigmasterol in the extract. These compounds increase NO production by macrophage cell lines. Low doses, 1–10 *μ*M, of hexadecanoic acid, octadecanoic acid, and linoleic acid enhanced NO production by J774 cells [62]. Stigmasterol-treated RAW264.7 cells produced elevated levels of NO compared to the control [63].

This study revealed the capacity of *T. brownii* stems bark DCM extract to elevate TNF-*α* levels in macrophages isolated from pyrogallol-immunosuppressed mice. This finding shows the immunostimulatory effect of the extract on innate immune responses via polarizing macrophages into classically-activated macrophages (M1 macrophages) [61]. TNF-*α* is a proinflammatory cytokine involved in the initiation of the expression of adhesion molecules on neutrophils and endothelial cells, leukocyte activation, and leukocyte chemotaxis [64].

The observed capacity of *T. brownii* DCM extract to augment the production of TNF-*α* by macrophages was attributed to the presence of hexadecanoic acid, octadecanoic acid, *β*-sitosterol, campesterol, and stigmasterol in the extract. These compounds augment TNF-*α* production in macrophages. Hexadecanoic acid and octadecanoic acid significantly enhanced the production of TNF-*α* by J774 cells [65]. *β*-sitosterol significantly increased TNF-*α* production by THP-1 macrophages [66]. Hexadecanoic acid was found to activate J774A.1 macrophages classically; it significantly augmented TNF-*α* levels [67]. The treatment of murine peritoneal macrophages with campesterol resulted in elevated levels of TNF-*α* compared with the control [68]. RAW264.7 cells treated with stigmasterol produced elevated levels of TNF-*α* in contrast to the untreated cells [63].

Our toxicity studies showed that *T. brownii* DCM extract is safe since it did not cause death or toxicity signs in rats. The acute toxicity of the extract was tested at 2000 mg/kg BW. Since it did not cause any harmful effect in rats, the LD_50_ of the extract was concluded to be above 2000 mg/kg, which is a wide safety margin [69]. Subacute toxicity study results further highlight the safety of *T. brownii* stem bark DCM extract.

## 5. Conclusions

*Terminalia brownii* stem bark DCM extract stimulated nonspecific immunity in pyrogallol-immunosuppressed mice by augmenting the production of three key elements of innate immunity; leukocytes, NO, and TNF-*α*. Our findings denote that *T. brownii* is a prospective candidate for developing new immunoenhancing medication for managing immunosuppressive ailments. GC-MS analysis of *T. brownii* stem bark DCM extract resulted in the identification of compounds shown to augment the production of leukocytes, NO, and TNF-*α*. Therefore, our study concludes that the observed immunostimulatory effect of the extract was due to the identified compounds in the extract. The DCM extract of *T. brownii* was safe since it did not cause any toxicity *in vivo* or *in vitro*.

## 6. Limitations of the Study

The study conducted only quantitative phytochemical analysis of the *T. brownii* DCM extract; the active phytochemicals were not isolated. Furthermore, a chronic toxicity test of the extract was not conducted.

## 7. Future Prospects

Isolation of various phytochemicals from the DCM extract of *T. brownii* and testing them for immunomodulatory activity. Additionally, assessment of chronic toxicity of the extract.

## Figures and Tables

**Table 1:** Composition of the standard rodent pellet feed.

*Composition*		
Corn	Fishmeal	Yeasts
Wheat	Hazelnut skin	Soya bean oil
Corn gluten feed	Calcium carbonate	Whey powder
Soya bean dehulled	Dicalcium phosphate	Sodium chloride

*Analytical components and supplements (minerals and vitamins)*		
18.50% protein	E671 (vitamin E)	E5 (Mn)
12% moisture	E672 (vitamin A)	E4 (Cu)
7% crude ash, 6% crude fibres	E1 (Fe)	E6 (Zn)
3% fats	E3 (Co)	E2 (I)

**Table 2:** Effect of *T. brownii* stem bark DCM extract on total and differential leukocyte counts.

Group	Leukocyte count (10^9/L)					
	WBCs	Neutrophils	Lymphocytes	Monocytes	Eosinophils	Basophils
Normal control	5.95 ± 0.11^*a*^	2.50 ± 0.07^*a*^	2.82 ± 0.06^*a*^	0.35 ± 0.02^*a*^	0.20 ± 0.01^*a*^	0.07 ± 0.00^*a*^
Negative control	0.91 ± 0.09^*d*^	0.40 ± 0.05^*e*^	0.48 ± 0.04^*d*^	0.01 ± 0.00^*d*^	0.01 ± 0.00^*d*^	0.01 ± 0.00^*d*^
Levamisole	6.02 ± 0.18^*a*^	2.62 ± 0.11^*a*^	2.82 ± 0.06^*a*^	0.31 ± 0.02^*b*^	0.20 ± 0.01^*a*^	0.07 ± 0.00^*a*^
TD (50 mg/kg BW)	2.70 ± 0.07^*c*^	1.07 ± 0.08^*d*^	1.42 ± 0.05^*c*^	0.13 ± 0.00^*c*^	0.06 ± 0.00^*c*^	0.03 ± 0.00^*c*^
TD (100 mg/kg BW)	3.14 ± 0.05^*c*^	1.43 ± 0.06^*c*^	1.46 ± 0.05^*c*^	0.16 ± 0.00^*c*^	0.07 ± 0.00^*c*^	0.03 ± 0.00^*c*^
TD (150 mg/kg BW)	4.63 ± 0.09^*b*^	2.03 ± 0.07^*b*^	2.28 ± 0.04^*b*^	0.16 ± 0.00^*c*^	0.12 ± 0.01^*b*^	0.04 ± 0.00^*b*^

Values were expressed as mean ± SEM. Statistical comparison was made within a column, and values with a similar superscript were not significantly different by ANOVA, followed by Tukey's post hoc test (*p* > 0.05). TD: *T. brownii* DCM extract.

**Table 3:** Effect of *T. brownii* stem bark DCM extract on the viability of vero cells and macrophages.

Group	% viability	
	Vero cells	Macrophages
Control	99.6 ± 0.1^*a*^	99.6 ± 0.1^*a*^
TD (46.88 *μ*g/ml)	93.9 ± 0.3^*b*^	93.4 ± 0.4^*b*^
TD (93.75 *μ*g/ml)	92.2 ± 0.3^*c*^	91.9 ± 0.2^*b*^
TD (187.5 *μ*g/ml	88.6 ± 0.3^*d*^	88.8 ± 0.3^*c*^
TD (375 *μ*g/ml)	83.3 ± 0.4^*e*^	80.7 ± 0.6^*d*^
TD (750 *μ*g/ml)	72.4 ± 0.1^*f*^	70.3 ± 0.4^*e*^
TD (1500 *μ*g/ml)	64.8 ± 0.3^*g*^	61.6 ± 0.3^*f*^
TD (3000 *μ*g/ml)	60.2 ± 0.2^*h*^	57.3 ± 0.3^*g*^

Values were expressed as mean ± SEM. Values with similar lowercase superscripts down a column are not significantly different by ANOVA followed by Tukey's post hoc test (*p* > 0.05). TD: *T. brownii* DCM extract.

**Table 4:** Effect of *T. brownii* stem bark DCM extract on the production of NO by mouse macrophages.

Group	Nitrite concentration (*μ*M)
Control	2.2 ± 0.2^*g*^
Levamisole	53.5 ± 0.5^*a*^
TD (46.88 *μ*g/ml)	2.4 ± 0.2^*g*^
TD (93.75 *μ*g/ml)	4.1 ± 0.5^*fg*^
TD (187.5 *μ*g/ml)	5.8 ± 0.5^*f*^
TD (375 *μ*g/ml)	10.0 ± 0.4^*e*^
TD (750 *μ*g/ml)	15.6 ± 0.8^*d*^
TD (1500 *μ*g/ml)	22.4 ± 0.8^*c*^
TD (3000 *μ*g/ml)	37.7 ± 0.6^*b*^

Values were expressed as mean ± SEM. Values with similar lowercase superscripts down a column are not significantly different by ANOVA followed by Tukey's post hoc test (*p* > 0.05). TD: *T. brownii* DCM extract.

**Table 5:** Effect of *T. brownii* stem bark DCM extract on TNF-*α* production by mouse macrophages.

Group	TNF-*α* concentration (pg/ml)
Control	97.3 ± 18.0^*h*^
Levamisole	2855.7 ± 23.8^*a*^
TD (46.88 *μ*g/ml)	915.0 ± 33.4^*g*^
TD (93.75 *μ*g/ml)	1042.0 ± 23.4^*g*^
TD (187.5 *μ*g/ml)	1294.7 ± 20.5^*f*^
TD (375 *μ*g/ml)	1499.0 ± 9.45^*e*^
TD (750 *μ*g/ml)	1765.7 ± 36.4^*d*^
TD (1500 *μ*g/ml)	2006.7 ± 36.5^*c*^
TD (3000 *μ*g/ml)	2388.0 ± 35.2^*b*^

Values were expressed as mean ± SEM. Values with similar lowercase superscripts down a column are not significantly different by ANOVA followed by Tukey's post hoc test (*p* > 0.05). TD: *T. brownii* DCM extract.

**Table 6:** Effect of *T. brownii* stem bark DCM extract on female Wistar rats' body weights (g).

Days	Normal control	TD (2000 mg/kg BW)
0	208.4 ± 2.7	205.4 ± 3.2
7	216.6 ± 3.0	214.0 ± 2.9
14	222.4 ± 2.7	219.0 ± 4.1

Statistical comparison was made within a column, and values were expressed as mean ± SEM. Values were not significantly different by one-way ANOVA (*p* > 0.05). TD: *T. brownii* DCM extract.

**Table 7:** Effect of *T. brownii* stem bark DCM extract on female Wistar rats' behaviour and overall appearance.

Behaviour and appearance	Observation	
	Normal control	TD (2000 mg/kg BW)
Sleep	Regular	Regular
Lethargy	Not present	Not present
Salivation	Not present	Not present
Tremors	Not present	Not present
Coma	Not present	Not present
Diarrhea	Not present	Not present
Convulsions	Not present	Not present
Alterations in the eyes, mucous membranes, skin, and fur	None	None

TD: *T. brownii* DCM extract.

**Table 8:** Effects of *T. brownii* stem bark DCM extract on female Wistar rats' body weights.

Days		0	7	14	21	28
Treatment	Normal control	207.4 ± 2.2	215.4 ± 2.4	221.2 ± 3.0	225.6 ± 3.3	229.0 ± 3.2
	TD (300 mg/kg BW)	207.6 ± 2.4	218.6 ± 2.3	226.8 ± 2.89	232.2 ± 2.6	235.60 ± 2.5
	TD (520 mg/kg BW)	210.4 ± 3.7	221.4 ± 3.1	230.4 ± 2.91	236.0 ± 2.8	239.40 ± 2.9
	TD (900 mg/kg BW)	208.6 ± 3.2	220.0 ± 2.8	228.8 ± 2.48	234.4 ± 3.4	238.40 ± 3.3

Values were expressed as mean ± SEM. Statistical comparison was made along rows, and values were not significantly distinct by one-way ANOVA (*p* > 0.05). TD: *T. brownii* DCM extract.

**Table 9:** Effect of *T. brownii* stem bark DCM extract on female Wistar rats' relative organ weights.

Organs	Normal control	TD (300 mg/kg BW)	TD (520 mg/kg BW)	TD (900 mg/kg BW)
Lungs	0.59 ± 0.03	0.58 ± 0.03	0.58 ± 0.04	0.57 ± 0.03
Spleen	0.41 ± 0.05	0.44 ± 0.06	0.41 ± 0.08	0.43 ± 0.06
Brain	0.73 ± 0.04	0.73 ± 0.02	0.71 ± 0.03	0.75 ± 0.03
Liver	3.53 ± 0.11	3.49 ± 0.19	3.41 ± 0.15	3.38 ± 0.16
Kidney	0.64 ± 0.03	0.63 ± 0.02	0.60 ± 0.02	0.61 ± 0.02
Heart	0.40 ± 0.01	0.37 ± 0.02	0.39 ± 0.01	0.37 ± 0.02

Values were expressed as mean ± SEM. Statistical comparison was made along rows, and values were not significantly distinct by one-way ANOVA (*p* > 0.05).

**Table 10:** Effect of *T. brownii* stem bark DCM extract on haematological parameters of female Wistar rats.

Parameter	Treatment			
	Normal control	TD (300 mg/kg BW)	TD (520 mg/kg BW)	TD (900 mg/kg BW)
White blood cells (10^∧^3/*μ*l)	8.99 ± 0.23	8.83 ± 0.31	8.94 ± 0.29	8.86 ± 0.25
Neutrophils (10^∧^3/*μ*l)	1.68 ± 0.06	1.67 ± 0.05	1.65 ± 0.05	1.62 ± 0.05
Lymphocytes (10^∧^3/*μ*l)	6.37 ± 0.11	6.24 ± 0.22	6.35 ± 0.16	6.32 ± 0.19
Monocytes (10^∧^3/*μ*l)	0.85 ± 0.03	0.83 ± 0.05	0.84 ± 0.03	0.83 ± 0.04
Eosinophils (10^∧^3/*μ*l)	0.07 ± 0.004	0.07 ± 0.004	0.07 ± 0.002	0.07 ± 0.003
Basophils (10^∧^3/*μ*l)	0.02 ± 0.004	0.02 ± 0.002	0.03 ± 0.002	0.02 ± 0.003
Erythrocytes (10^∧^6/*μ*l)	7.72 ± 0.26	7.69 ± 0.26	7.81 ± 0.17	7.48 ± 0.34
Hemoglobin (g/dl)	13.79 ± 0.62	13.44 ± 1.01	13.68 ± 0.79	13.74 ± 0.71
Hematocrit (%)	43.10 ± 3.75	41.84 ± 3.59	39.76 ± 3.05	44.54 ± 5.25
Mean corpuscular volume (fl)	55.48 ± 3.67	54.90 ± 2.81	56.10 ± 3.29	53.46 ± 3.47
Mean corpuscular hemoglobin (pg)	21.60 ± 2.66	23.02 ± 1.87	22.98 ± 2.04	20.82 ± 1.84
Red cell distribution width (%)	16.12 ± 2.77	14.60 ± 1.29	15.36 ± 1.69	17.04 ± 1.45
Platelets (10^∧^3/*μ*l)	728.60 ± 19.50	746.80 ± 25.50	713.80 ± 29.10	716.80 ± 23.10
Mean platelet volume (fl)	7.62 ± 0.81	7.38 ± 1.02	7.22 ± 0.79	7.60 ± 0.97

TD: *T. brownii* DCM extract.

**Table 11:** Effect of *T. brownii* stem bark DCM extract on female Wistar rats' biochemical parameters.

Parameter	Treatment			
	Normal control	TD (300 mg/kg BW)	TD (520 mg/kg BW)	TD (900 mg/kg BW)
Potassium ions (mmol/L)	4.7 ± 0.3	4.9 ± 0.2	4.7 ± 0.2	4.7 ± 0.2
Chloride ions (mmol/L)	105.6 ± 1.2	103.3 ± 4.4	104.4 ± 5.1	101.5 ± 4.4
Sodium ions (mmol/L)	141.8 ± 3.5	142.9 ± 4.6	143.6 ± 3.6	138.2 ± 3.7
Creatine (*μ*mol/L)	51.7 ± 2.4	50.6 ± 2.2	48.8 ± 2.3	51.0 ± 2.6
Urea (mmol/L)	6.2 ± 0.3	6.0 ± 0.2	6.5 ± 0.2	6.1 ± 0.2
Alkaline phosphatase (U/L)	132.6 ± 6.7	144.0 ± 7.4	135.2 ± 4.9	139.8 ± 3.4
Alanine transaminase (U/L)	69.3 ± 4.0	69.9 ± 3.6	64.1 ± 4.2	61.7 ± 3.3
Aspartate transaminase (U/L)	108.6 ± 7.3	110.0 ± 4.3	104.2 ± 4.0	101.0 ± 5.3
Bilirubin (*μ*mol/L)	2.9 ± 0.1	3.0 ± 0.1	2.9 ± 0.1	2.7 ± 0.1
Albumin (g/L)	35.1 ± 2.6	36.1 ± 2.0	29.6 ± 1.6	37.4 ± 2.3
Globulin (g/L)	35.7 ± 1.7	33.1 ± 1.8	37.4 ± 1.6	30.3 ± 2.4
Total protein (g/L)	64.0 ± 2.4	69.8 ± 2.0	61.4 ± 2.2	65.8 ± 2.0
Glucose (mmol/L)	7.0 ± 0.3	7.1 ± 0.4	8.1 ± 0.6	8.1 ± 0.5
Cholesterol (mmol/L)	1.9 ± 0.1	1.9 ± 0.1	1.8 ± 0.1	1.8 ± 0.1

TD: *T. brownii* stem bark DCM extract.

**Table 12:** Compounds detected in stem bark DCM extract of *T. brownii* through GC-MS.

RT	Compound name	Class	Molecular formula	Conc. (*μ*g/g)	% abundance
23.71	Hexadecanoic acid	Fatty acid	C_16_H_32_O_2_	14.61	30.11
25.35	Linoleic acid	Fatty acid	C_18_H_32_O_2_	5.01	10.32
25.56	Octadecanoic acid	Fatty acid	C_18_H_36_O_2_	3.05	6.28
31.05	Squalene	Triterpenoid	C_30_H_50_	0.10	0.20
37.43	Campesterol	Steroid	C_28_H_48_O	0.30	0.61
37.58	Stigmasterol	Steroid	C_29_H_48_O	0.07	0.14
38.77	Beta-sitosterol	Steroid	C_29_H_50_O	0.37	0.76

## Data Availability

The data supporting the authors' conclusions are available in this article.
